# Residual Strain Development in Rapid Frontally Curing Polymers

**DOI:** 10.1021/acsaenm.4c00526

**Published:** 2024-10-31

**Authors:** Zhuoting Chen, Behrad Koohbor, Xiang Zhang, Leon M. Dean, Philippe H. Geubelle, Nancy R. Sottos

**Affiliations:** † Mechanical Engineering Department, University of Wyoming, Laramie, Wyoming 82071, United States; ‡ Department of Mechanical Engineering, Rowan University, Glassboro, New Jersey 08028, United States; § Beckman Institute for Advanced Science and Technology, University of Illinois at Urbana-Champaign, Urbana, Illinois 61801, United States; ∥ Department of Materials Science and Engineering, University of Illinois at Urbana-Champaign, Urbana, Illinois 61801, United States; ⊥ Department of Aerospace Engineering, University of Illinois at Urbana-Champaign, Urbana, Illinois 61801, United States

**Keywords:** frontal polymerization, process-induced residual deformation, thermo−chemo−mechanical model, dicyclopentadiene (DCPD), digital image correlation

## Abstract

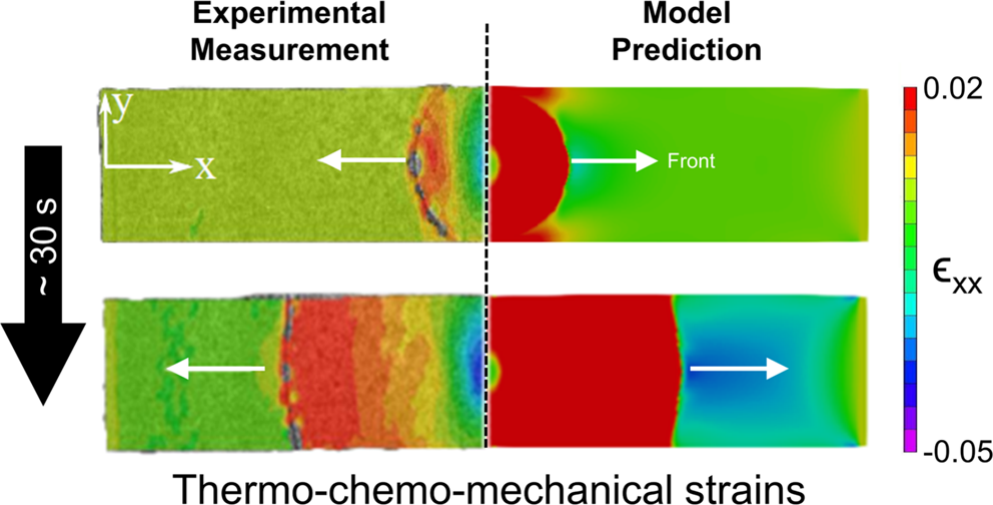

Frontal polymerization (FP) has emerged as a rapid and energy-efficient process for fabricating thermoset polymers and composites. In this process, a self-propagating reaction front cures the polymer rapidly by the exothermic heat of polymerization reaction instead of an external heat source. Design for FP-based manufacturing in commercial applications requires more comprehensive characterization and prediction of material evolution and residual deformation throughout the process. Here, we report experimental and numerical studies in response to this need. The experimental study focuses on measuring the temperature and cure-dependent properties of mono/poly dicyclopentadiene to capture the strain evolution during the frontal polymerization process. The experimentally measured elastic moduli, Poisson’s ratios, and coefficients of thermal expansion and chemical shrinkage show strong dependence on the degree of cure. Based on the experimental output, a coupled thermo–chemo–mechanical model has been developed to capture the measured residual strains. The chemical shrinkage is closely related to the curing rate, leading to strong localization of residual strains in accelerated reaction regions, especially where two fronts merge. Preheating of the monomer (or gel) at the fronts merging area is suggested as an effective method to mitigate residual deformations.

## 1 Introduction

Frontal polymerization (FP) stands out as a rapid and energy-efficient manufacturing process for the fabrication of thermosetting polymers.^1,2^ Curing of thermoset polymers by frontal polymerization involves a self-propagating exothermic reaction in a monomer, gel, or mixture of monomers triggered by an external stimulus, such as heat, light, or a chemical initiator. It has been documented that the frontal polymerization of dicyclopentadiene (DCPD) enables the fabrication of high-performance polymeric and composite parts with thermo-mechanical properties comparable to aerospace-grade thermosets.^1,3,4^ The ability to extend the room-temperature liquid-processing window of DCPD by appropriate inhibitors, the possibility of developing viscoelastic gels with tunable rheological profiles, and the inclusion of various fillers into the solution have led to the use of FP-cured DCPD in direct ink write 3D printing,^5−7^ energy-efficient manufacturing of composites,^8−10^ concurrent curing and vascularization,^11−13^ and the development of thermosets with patterns^14,15^ and graded thermo-mechanical properties.^16−18^

Comparable to conventional curing techniques (e.g., autoclave), FP entails concomitant thermal, chemical, and mechanical changes occurring within the material throughout the polymerization phase. Curing of thermosets is associated with volume changes caused by chemical shrinkage and thermal deformations due to heating/cooling cycles. While chemical shrinkage is dependent on the degree of cure (α) of the thermoset, thermal deformations are complex functions of α and the glass transition temperature of the material.^19−21^ Regardless of their origin, the combined effects of these two phenomena often lead to the development of residual deformations in the fully cured polymer. If mechanically constrained by rigid mold walls, these residual deformations result in residual stresses that can be detrimental to the service life of the product.^5^

Model predictions of process-induced residual stresses in conventionally cured thermosets have been discussed in numerous studies. Multiscale thermo–chemo–mechanical models have been developed and used to predict cure-dependent residual deformations in conventional curing of thermoset polymers and polymer composites.^22,23^*In-situ* measurements have complemented and validated model predictions. Noncontact, image-based techniques have been utilized to measure cure-dependent shrinkage and thermal expansion of thermosets,^19,24−26^ as well as the curing-induced residual deformations in various thermosetting polymer composites.^28^

More complex process-induced residual deformations occur during frontal polymerization compared to autoclave-based curing processes due to the highly localized chemical reaction and exothermic heat generated as the front moves through the material.^29^ Both the degree of cure and the temperature vary temporally and spatially in frontal polymerization. Sharp thermal and α gradients across the polymerization front give rise to heterogeneous thermo-mechanical strain fields.^1,8^ Mechanical (e.g., Young’s modulus, Poisson’s ratio) and thermo-physical (e.g., coefficient of thermal expansion) properties of most thermosetting polymers, including DCPD, vary with temperature and α.^29,30^ Therefore, considering the rapidly evolving and highly heterogeneous temperature and α fields, it is difficult to track the evolution of all thermo-mechanical properties in time and space. Further, the energy efficiency of the FP curing process can be improved by using multiple triggering points.^1,12^ Upon merging of multiple fronts, steeper thermal gradients will develop, leading to the evolution of even more heterogeneous deformation fields in the vicinity of the fronts merging area.

Understanding the evolution of material properties and the accumulation of residual stresses during the process is crucial for enhancing the precision of FP-based manufacturing. FP is inherently a multiphysics process that involves coupled heat conduction, chemical reaction, and mechanical deformations. A fully coupled thermo–chemo–mechanical model requires simultaneous solutions for temperature, α, and displacement fields. Since the deformation response is driven by thermal expansion and chemical shrinkage during the polymerization process, a more efficient modeling strategy is to first determine the temperature and the α history, and then feed the output to a mechanics-based model to compute the corresponding mechanical response. Temperature and α evolutions can be obtained either by prescribing a temperature history to quantify the curing response^19,28,31−33^ or by a fully coupled thermo-chemical simulation.^23,34,35^ The latter method alleviates the potential numerical difficulties associated with simultaneous solutions of the three fields (i.e., chemical, thermal, and mechanical). Additionally, it streamlines the material calibration process by initially focusing on calibrating parameters associated with the thermal and chemical aspects before addressing those related to the constitutive laws.

The coupled thermo-chemical process that captures the response of a self-propagating front in the FP process has been investigated for pure DCPD^9^ as well as for carbon and glass fiber-reinforced composites.^8,36,37^ In addressing the mechanical behavior of the system, the primary focus lies in formulating cure- and temperature-dependent mechanical constitutive laws to capture the rapidly changing material properties during the curing process. Different constitutive laws have been employed to characterize the behavior of polymers and polymer composites as summarized in Kim et al.^38^ For example, Zhu and Geubelle^33^ and Kim and White^38^ investigated the residual deformation in thermosetting composites during the oven-curing process using a viscoelastic constitutive law, wherein extensive stress relaxation tests were conducted to obtain the corresponding Prony series representation. Similar viscoelastic constitutive laws were used by different researchers to model process-induced residual deformations.^23,31^ The cure hardening instantaneously linear elastic (CHILE) model has also been widely used for characterizing process-induced residual deformations with reasonable accuracy.^28,39^ Kumar et al.^40^ investigated surface pattern formation induced by oscillatory loading in frontally polymerizing gels. In another recent report, Kumar et al.^41^ modeled the intricate relationship between extrusion speed, printing kinetics, and material deformation in FP-based additive manufacturing. By exploring the interplay among these factors, they offered pivotal insights for optimizing printing parameters in additive manufacturing, aiming to enhance structural integrity and mechanical performance.

The objective of the present work is to develop a hybrid, multiphysics experimental and modeling approach to predict thermo–chemo–mechanical fields during frontal polymerization. Thermo-mechanical properties of DCPD are first characterized at different cure and temperature conditions. Digital image correlation (DIC) is used to measure the volumetric changes at different temperatures and cure conditions as well as α-dependent elastic moduli and Poisson’s ratios in DCPD. These properties serve as inputs to a fully coupled CHILE model to simulate the residual deformation fields and curing rate-dependent chemical shrinkage. Furthermore, experimental and modeling characterizations are performed to visualize the effects of boundary conditions and multifront FP process on the development of residual strain fields in DCPD samples. This manuscript is structured as follows: Experiments designed for the characterization of cure and temperature-dependent properties as well as those used for quantitative study of the effects of boundary conditions are outlined in Section 2. Section 3 details the multiphysics modeling approach. Section 4 compares the strain fields obtained from experiments and simulations and describes potential methods for mitigating process-induced deformations in FP using the implemented computational model.

## 2 Experimental Methods

### 2.1 DCPD Gel Preparation

Dicyclopentadiene (DCPD), 5-ethylidene-2-norbornene (ENB), second generation Grubbs’ catalyst (GC2), and phenylcyclohexane (PCH) were purchased from Sigma-Aldrich. Since DCPD is solid at room temperature, 5 wt % ENB was added to depress the melting point below room temperature and facilitate handling. Herein DCPD refers to this 95:5 DCPD:ENB mixture. Tributyl phosphite (TBP) inhibitor was purchased from TCI Chemicals and stored under inert gas. All other chemicals were used as received.

Liquid resin was prepared by mixing the appropriate amount of DCPD with GC2 and TBP, using PCH as a solvent. For a typical sample, GC2 (12.5 mg, 14.7 μmol) was dissolved in PCH (625 μL) using bath sonication (10 min). TBP (4.0 μL, 14.7 μmol, 1 equiv) was added, and the solution was then thoroughly mixed with DCPD (19.46 g, 147 mmol, 10,000 equiv). The low-viscosity liquid resin was carefully poured into a rectangular cell casting mold consisting of borosilicate glass plates and a polyurethane spacer (3 mm thick). The resin was then held in an oven at 30 °C for a predetermined time to achieve a prescribed α value for the resulting gel, with 3 h corresponding to a degree of cure of approximately 0.28. Finally, the gel was removed from the mold and cut to size. Gel samples were stored in a freezer (ca. −20 °C) before testing.

### 2.2 Front Velocity Measurement

The velocity of propagating reaction fronts was measured by tracking the location of the front in time using a Canon EOS 7D digital camera, similar to the method described in Robertson et al.^1^ and Dean et al.^16^ The reaction front position was identified by the refractive index mismatch between the cured polymer and uncured monomer (or partially cured gel). Front velocity was determined as the slope of the best linear fit to the front location-time data for samples with various initial degrees of cure.

### 2.3 Digital Image Correlation

Strain fields were measured via DIC during polymerization front propagation and used to calculate the associated chemical shrinkage, coefficient of thermal expansion, and α-dependent mechanical parameters in DCPD samples. Partially cured gel samples were taken out of the freezer and left in ambient conditions for about 2 min until thermally stabilized. Samples with appropriate dimensions were extracted from partially cured 100 mm × 100 mm gel panels with a razor blade. The surface of the gel piece was spray-coated with a thin layer of flat white paint. Black speckle particles were immediately sprayed onto the wet white substrate. Black speckle pattern in this work was produced by suspending 5 vol % toner carbon powder (ca. 30 μm average particle diameter) in ethanol and sprayed directly on the sample surface by an airbrush. The DIC sample preparation took less than 3 min, which was short enough to ensure that the initial degree of cure of the gel samples remained nearly unchanged. In all DIC measurements, the strain noise floor was determined according to the procedures detailed in Koohbor et al.^42^ In all cases, the strain noise floor was approximately 300 × 10^–6^.

### 2.4 Characterizing Degree of Cure-Dependent Properties with DIC

The elastic modulus and Poisson’s ratio of gel samples at different degrees of cure were calculated from the front (camera-facing) surface of tensile gel coupons with dimensions of 60 mm × 10 mm × 3 mm. The speckle pattern for DIC was applied to these specimens using the approach described in Section 2.3. Two 10 mm × 10 mm grip sections at sample ends were fixed inside the grips of a table-top screw-driven tensile frame, which was equipped with a 50 N load-cell. The sample was then elongated at a constant rate of 0.1 mm/s. During loading, images were captured at a frequency of 2 Hz from the speckle-patterned surface of the gel piece. Tensile force, along with the axial and lateral strain components measured on the surface of the gel samples, were used to determine the elastic modulus and Poisson’s ratio. After unloading, the tensile sample was removed and stored in a temperature-controlled area (*T* = 21 ± 0.5 °C) for specific time periods to allow for nonfrontal curing (background reaction) to progress. As shown in Figure S1a of the Supporting Information document, the degree of cure of a gel sample can be tracked in time. Therefore, mechanical testing of gel samples at certain times enabled correlating tensile moduli and Poisson’s ratios of the samples with their degree of cure. The same loading-imaging process was repeated on the sample after certain time periods (corresponding to certain degrees of cure) to characterize the degree-of-cure-dependent tensile modulus and Poisson’s ratio. The repeatability of the mechanical data was confirmed by following the same test procedure for at least five independently prepared samples.

Chemical shrinkage of gel samples at different degrees of cure was characterized by measuring the temporal evolution of in-plane strains on the surface of a gel sample, curing at 21 ± 0.5 °C (nonfrontal). Images were captured from the surface of the gel sample at 30 min time intervals for a duration of 48 h. The chemical shrinkage of the DCPD gel sample was determined by linear correlation between curing-induced in-plane strain components and α.^27^ More details regarding the experimental measurement of chemical shrinkage are provided as Supporting Information.

The temperature-dependent coefficient of thermal expansion (*k*^t^) was measured for partially cured gel and fully cured polymers using two independent methods. The first method used DIC to determine a correlation between temperature and thermal strains for two cure conditions, i.e., DCPD gel with α = 0.28 and fully cured polymer. *k*^t^ of the fully cured polymer was determined by measuring the in-plane thermal strains in a freely expanding sample on a nominally frictionless flat fixture placed directly on a heating stage. The sample temperature was monitored by a noncontact IR thermometer. In-plane strains were measured at temperatures ranging from 20 to 200 °C. The slope of the best linear fit to the strain-temperature data was determined as the *k*^t^ value for the fully cured polymer. The application of a similar approach on a partially cured gel piece was not possible since the degree of cure in the gel could not be maintained constant during the heating process. To avoid measurement uncertainties associated with the concurrent temperature and α-dependent *k*^t^ in gel samples, *k*^t^ of gel was measured in low temperature conditions in the range of −20 to 0 °C, i.e., a temperature range wherein the degree of cure effectively remains constant. A gel sample with α = 0.28 was speckle patterned and placed on a custom-made fixture that was cooled to −20 °C by means of a dry ice-methanol solution. The sample temperature was increased to 0 °C by allowing natural heat convection. Images were captured from the speckled (exposed) surface of the gel at different temperatures. The sample temperature was monitored by a thermocouple. A linear correlation between DIC-measured strains and the temperature was used to determine *k*^t^ of the partially cured gel.

The *k*^t^ of gels at different degrees of cure was also characterized with an independent approach using a TA Instruments RSA-G2 dynamic mechanical analyzer (DMA) equipped with tension fixtures and a refrigerated cooling system. The degree of cure and *T*_g_ of the gel samples were measured by differential scanning calorimetry (DSC) (10 °C/min ramp rate). The gel panels described above were cut into specimens with dimensions of approximately 30 mm × 4 mm × 0.5 mm, cooled below their *T*_g_, and loaded into the DMA tension fixtures with an initial gap of 10 mm. The samples were then heated from −40 to 20 °C at 3 °C/min while the instrument continuously adjusted the gap to maintain zero axial force. The *k*^t^ was extracted from a best-fit line of the thermal strain vs temperature across linear regions, covering a range of at least 15 °C either below or above *T*_g_. The *k*^t^ of a frontally polymerized sample (degree of cure >90%) was also measured using the same procedure over a range of 20–200 °C to validate the values obtained by DIC.

### 2.5 Evolution of Strain and Temperature Fields during FP

**Figure 1:**
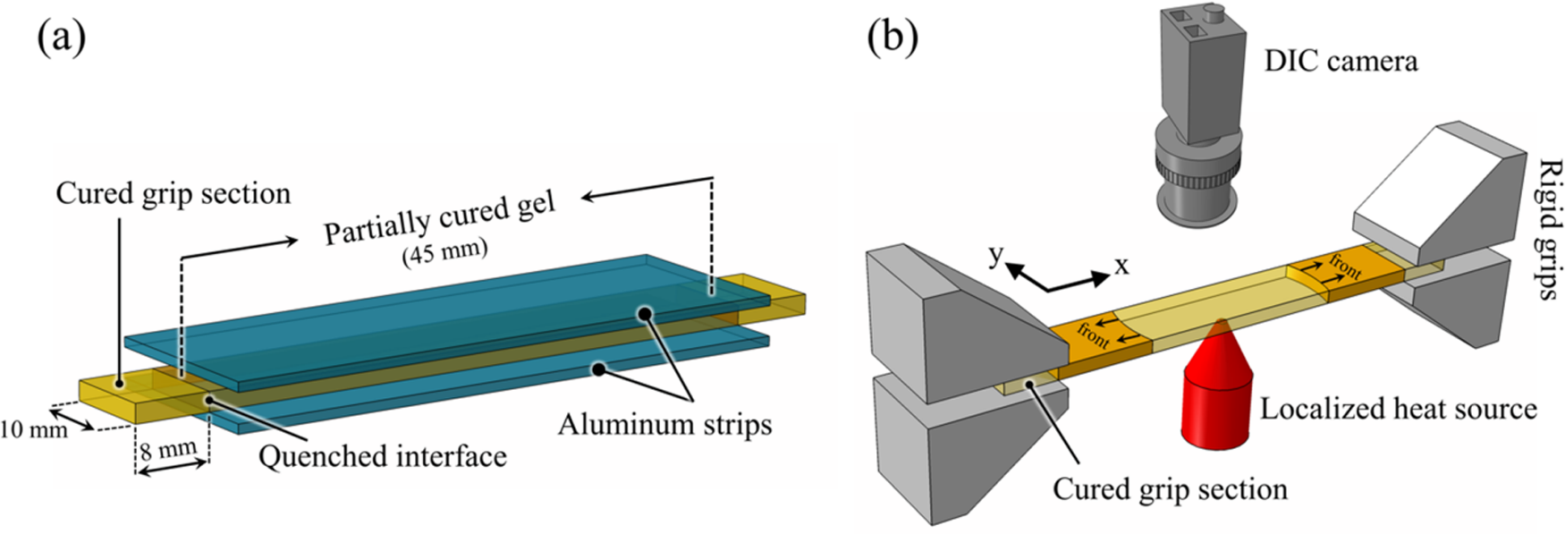
Schematics of (a) gel specimen with cured grip sections prepared by using aluminum strips as heat sink, and (b) DIC measurement setup developed for characterizing in-plane strain fields during the polymerization of the gel specimen affixed in a rigid frame.

The transient strain fields developed during frontal polymerization were characterized in gel specimens (initial α: 0.28) with clamped ends. Gel specimens for strain measurements were prepared as strips with their ends previously cured according to the following procedure. The middle section of a gel specimen (60 mm × 10 mm × 1.5 mm) was first sandwiched between two aluminum strips, and fronts were initiated at the two ends. The aluminum strips were positioned to serve as a heat sink to quench the front, leaving the partially cured gel piece in the middle area of the sample intact. Figure 1a shows the gel specimen with fully cured grip sections at either end. This specimen shows three distinct regions, with the middle portion made of DCPD gel (∼45 mm long) and the two ends that serve as grip sections made of fully cured stiff pDCPD. Between the two regions, there is a very thin region referred to as the quenched interface. Inside the quenched interface, the stiffness rapidly drops from that of fully cured pDCPD to a gel with a degree of cure of 0.28.

The cured ends of the specimen were then fixed inside two rigid grips in an Instron 8841 load frame, as shown in Figure 1b. The front side of the specimen was speckle patterned for DIC strain measurement. FP was retriggered by a local heat source (soldering iron) applied to the center of the rear side of the gel specimen. During FP, time-lapse images were captured at 2 Hz frequency. Image acquisition continued until the specimen was fully cured and then cooled to room temperature. Images were analyzed in the commercial DIC software Vic-2D (Correlated Solutions, Inc.) with subset, step, and strain filter sizes that were optimized for the highest strain-to-noise ratio while enabling the capture of sharp strain gradients across polymerization fronts.^42^

## 3 Multiphysics Modeling of the FP Process

**Figure 2:**
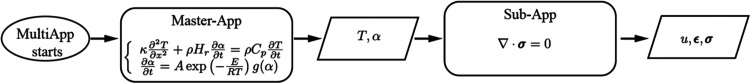
Schematic illustration of the thermo–chemo–mechanical modeling scheme.

The solution of the thermo–chemo–mechanical problem involved first solving the fully coupled thermo-chemical differential equations, and then transferring the degree of cure and temperature histories into a mechanics framework. This scheme, illustrated in Figure 2, was implemented in the open-source code Multiphysics Object-Oriented Simulation Environment (MOOSE)^43^ as a Multi-App simulation.^44^ In this Multi-App simulation, the coupled thermo-chemical problem was solved in the Master-App. The resultant temperature and degree of cure histories were then fed into a Sub-App where the mechanical problem was solved. Each of the two components is discussed in detail below. We should emphasize that the modeling approach presented here relies on several assumptions and simplifications, including small deformation assumption and the use of a simple cure hardening instantaneously linear elastic model rather than more accurate viscoelastic models. In addition, to enable direct comparison between experimental measurements and model predictions, we only focus on strain fields. Therefore, the present study does not report the evolution of stresses during frontal polymerization.

### 3.1 Coupled Thermo-Chemical Modeling of the FP Process

The polymerization process is described by the following coupled thermo-chemical equations^1,35^
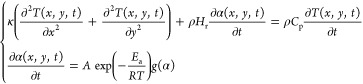
1where the first equation represents the thermal diffusion problem and the second denotes the cure kinetics model expressed in terms of the temporally and spatially dependent temperature *T* (in K) and degree of cure α fields. In the first equation, κ (in W/(m·K)) is the thermal conductivity, ρ (in kg/(m^3^)) is the density, *C*_p_ (in J/(kg·K)) is the specific heat, and *H*_r_ (in J/kg) is the total enthalpy of the reaction. The heat of reaction term in the first equation is related to the rate of cure expressed in the cure kinetics model by an Arrhenius relation characterized by the time constant *A* (in s^–1^), the activation energy *E*_a_ (in kJ/mol), the universal gas constant *R* (in J/(mol·K)), and a reaction model *g*(α(*x,y,t*)). In this work, the function *g*(α) takes the form
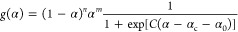
2where *n* and *m* are the exponents in the Prout-Tompkins autocatalytic model, *C* and α_c_ are nondimensional constants introduced to incorporate diffusion effects, and α_0_ denotes the initial value of the degree of cure, α. This model has been used to simulate the FP-based manufacturing process of DCPD monomer,^10,45^ gel,^46^ and composite systems.^8,9,12,13,47^ To determine the parameters in the cure kinetics model, a constrained nonlinear multivariable optimization algorithm^48^ was adopted to identify a set of parameters that best match multiple ramped DSC measurements. The fitting of the DSC data as well as the validation of the thermo-chemical model through comparison between simulated and experimentally measured front velocity at different initial degrees of cure are discussed in Section 4.

### 3.2 Cure and Temperature-Dependent Mechanical Properties

For the mechanical problem, we adopted the CHILE model^39^ and implemented an incremental elasticity formulation. In this formation, at analysis increment *n* + 1, the stress increment is updated using the instantaneous elastic moduli, *C*(α_*n*+1_), the total strain increment, Δϵ_*n*+1_, and the eigenstrain increments due to thermal expansion, Δϵ_*n*+1_^t^, and chemical shrinkage, Δϵ_*n*+1_^c^ as
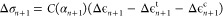
3where the thermal strain expansion and chemical shrinkage strain depend on the current and previous values of the temperature and degree of cure, respectively,i.e.,
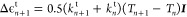
4
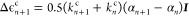
5In these equations, ***I*** is the identity tensor and *k*_*n*+1_^t^ and *k*_*n*+1_^c^ denote the instantaneous coefficients of thermal expansion and chemical shrinkage, respectively. The numerical values of these coefficients and their dependence on the temperature and degree of cure were determined using experimental measurements described in the forthcoming sections. Note that the thermal “expansion” and chemical “shrinkage” indicate volumetric variations with opposite signs. However, for simplicity, we show both the expansion and shrinkage coefficients in positive signs in our problem formulation. The numerical values corresponding to these coefficients are obtained and reported with appropriate signs, i.e., *k*^t^ in positive and *k*^c^ with negative values.

### 3.3 Simulation Domain of the Thermo–Chemo–Mechanical Process

**Figure 3:**
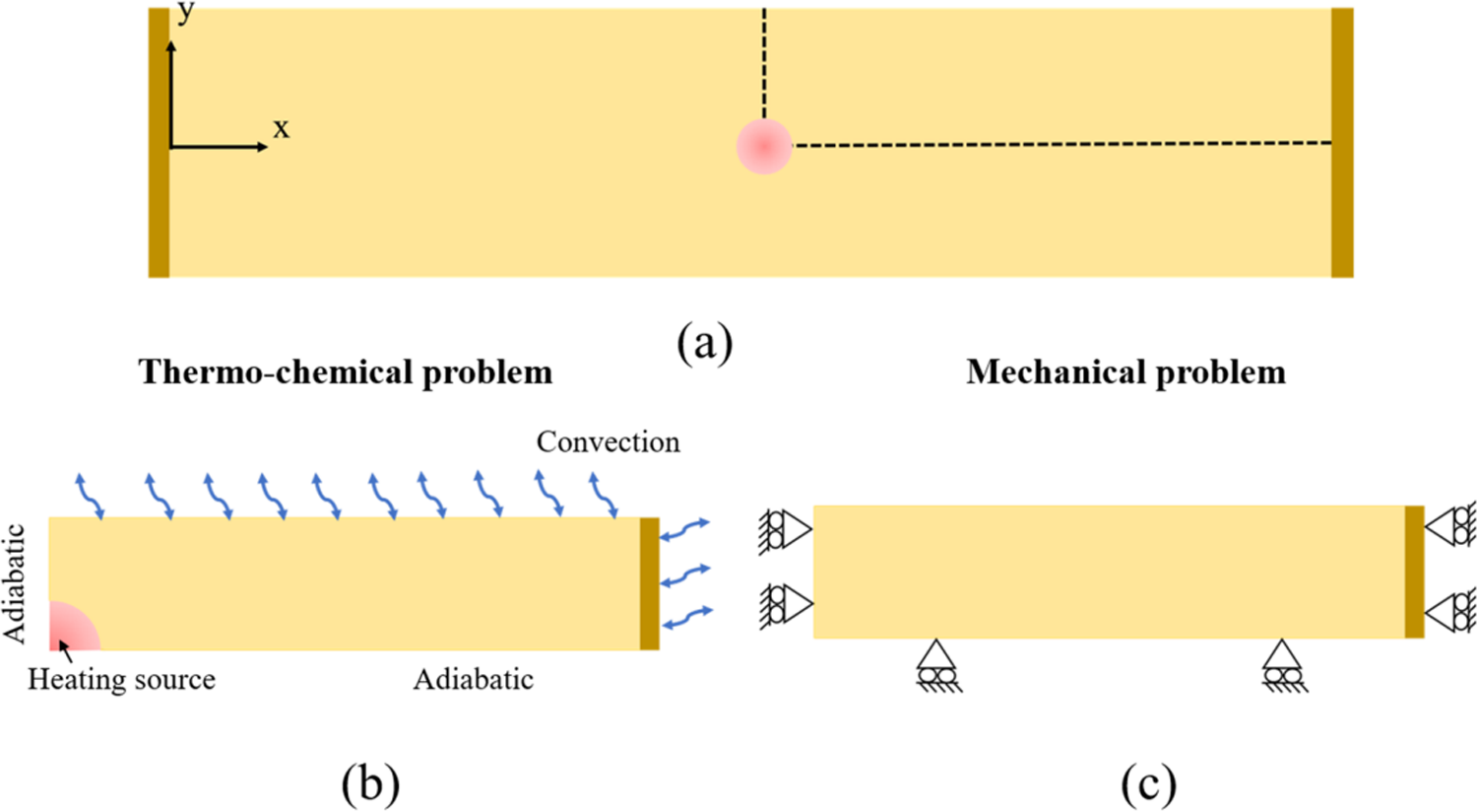
Single front initiated from the center of a double-clamped specimen: (a) simulation domain where only a quarter of the domain (dash line) was considered due to symmetry, (b) setup for the thermo-chemical problem, and (c) setup for the mechanical problem.

To determine the curing rate-dependent coefficient of chemical shrinkage (*k*^c^), we simulated the strain formation in the specimen shown in Figure 1. The 2D simulation domain only consisted of the gel and the interface region as shown in Figure 3a, where the thickness of the interface is taken to be 0.02 mm, consistent with the thickness of the front in DCPD.^9^ Due to symmetry, only a quarter of the domain was used for simulation. The boundary conditions used in the Master-App of the thermo-chemical simulation are shown in Figure 3b. Note that the convection heat loss on the front and back of the specimen could not be accounted for in the 2D simulations. Therefore, a heat sink term was included in the thermal diffusion equation in eq 1, leading to the following coupled thermo-chemical problem
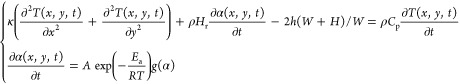
6where *W* and *H* are the width and thickness of the specimen, respectively. Parameter *h* denotes the film coefficient (=25 W/m^2^ K) and the ambient temperature was chosen to be 20 °C.^11^ All other parameters are the same as in eq 1.

The localized heat source (soldering iron) was represented by a circular area with a radius of 3 mm where the temperature reduces linearly from 200 °C at the center to 170 °C at the edge. The heating was applied for 1 s to initiate the front.

The temperature and degree of cure evolutions computed in the Master-App simulation serve as input for an accompanying Sub-App simulation. Utilizing the same mesh, temperature profiles, degree of cure history, and curing rates, the Sub-App conducted a mechanical analysis to determine the resulting mechanical deformations.

## 4 Results and Discussion

### 4.1 Capturing the Thermo-Chemical Process

**Figure 4:**
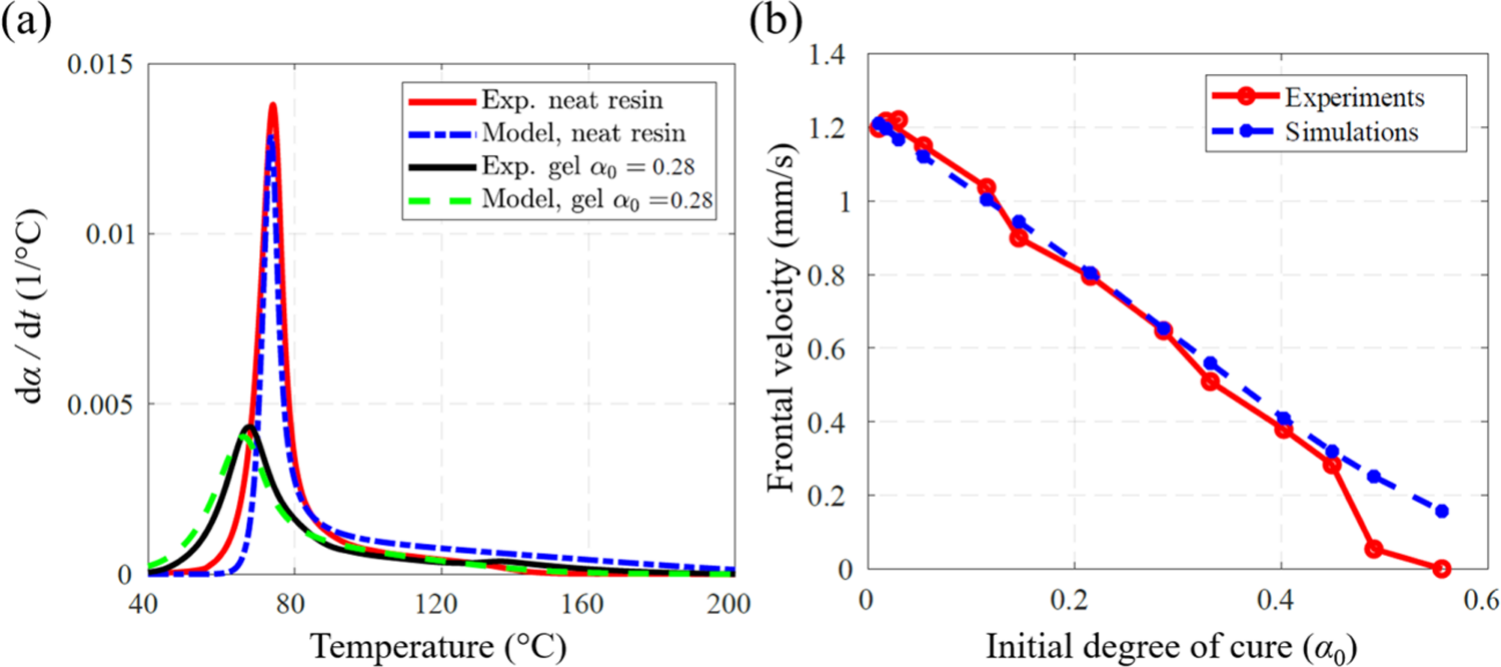
Calibration and validation of the cure kinetics for different initial degree-of-cure conditions: (a) DSC fitting using the data from neat resin and a gel with an initial degree of cure of 0.28; (b) comparison between measured and simulated front velocities at different values of the initial degree of cure.

Figure 4a presents the experimental and fitted DSC curves for neat resin and a gel with α_0_ = 0.28. The cure kinetic parameters, *H*_r_, *A, E*_a_, *m*, and *n* in eqs 1 and 2 were determined from the fitted DSC curves as described in our prior work.^1,8−10^ The cure kinetics parameters and thermo-mechanical properties used in the simulations are listed in Tables 1 and 2, respectively. Using the calibrated cure kinetics model and the material parameters in these tables, thermo-chemical simulations were conducted at different initial degrees of cure. The computed front velocities were compared with experimental values, showing a good agreement as depicted in Figure 4b.

**Table 1:** Cure Kinetics Parameters Used in the Reaction-Diffusion Model

*E*_a_ (kJ mol^–1^)	*A* (s^–1^)	*m*	*n*	*C*	α_c_
113.2	8.5 × 10^15^	0.78	1.74	15.2	0.36

**Table 2:** Material Properties Used in the Thermo-Mechanical Model^6^

*H*_r_ (J/g)	κ (W m^–1^K^–1^)	ρ (kg m^–3^)	*C*_p_ (J)
370	0.15	980	1600

### 4.2 Cure-Dependent Modulus and Poisson’s Ratio

**Figure 5:**
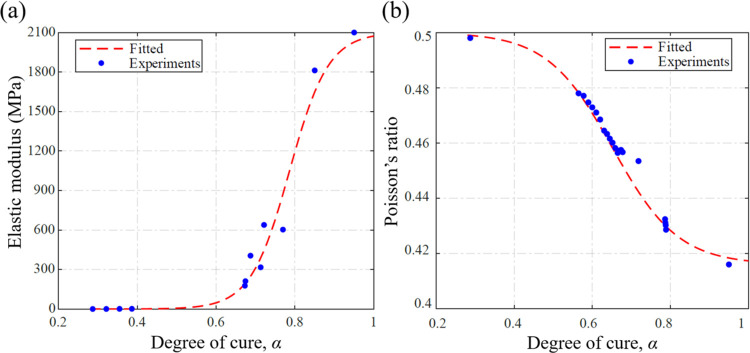
Variation of (a) tensile elastic modulus and (b) Poisson’s ratio with degree of cure. Data in these graphs are obtained from measurements performed on multiple gel samples.

Figure 5 shows the dependence of the tensile modulus and Poisson’s ratio of DCPD on the degree of cure α. The modulus of the gel samples with α < 0.4 was less than 2 MPa. The modulus increases significantly when α exceeds 0.6, reaching a maximum value of 2.1 GPa for a fully cured polymer. An inverse trend was observed for Poisson’s ratio, which decreased from 0.49 for α = 0.28–0.41 for a fully cured polymer. The values for the tensile modulus and Poisson’s ratio of fully cured DCPD samples were consistent with previously reported data.^16,49^ These measurements were fitted with sigmoidal functions and used as input to the multiphysics model. The α-dependence of the elastic modulus (Figure 5a) was modeled as
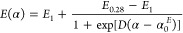
7where *E*_1_ (=2.1 GPa) and *E*_0.28_ (=290 kPa) respectively denote the moduli of DCPD at degrees of cure of 1.0 (fully polymerized) and 0.28. *D* = 20.3 and α_0_^*E*^ = 0.79 are the two nondimensional parameters controlling the shape of the sigmoidal function. A similar function was used to fit the α-dependence of the Poisson’s ratio (Figure 5b)
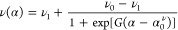
8where *ν*_1_ = 0.416 and *ν*_0_ = 0.5 represent the Poisson’s ratios of DCPD at α = 1.0 and 0.28, respectively, while the parameters *G* = 11.92 and α_0_^υ^ = 0.65 govern the general shape of the fitting function.

### 4.3 Degree of Cure and Temperature Dependence of Chemical Shrinkage and *k*^t^

**Figure 6:**
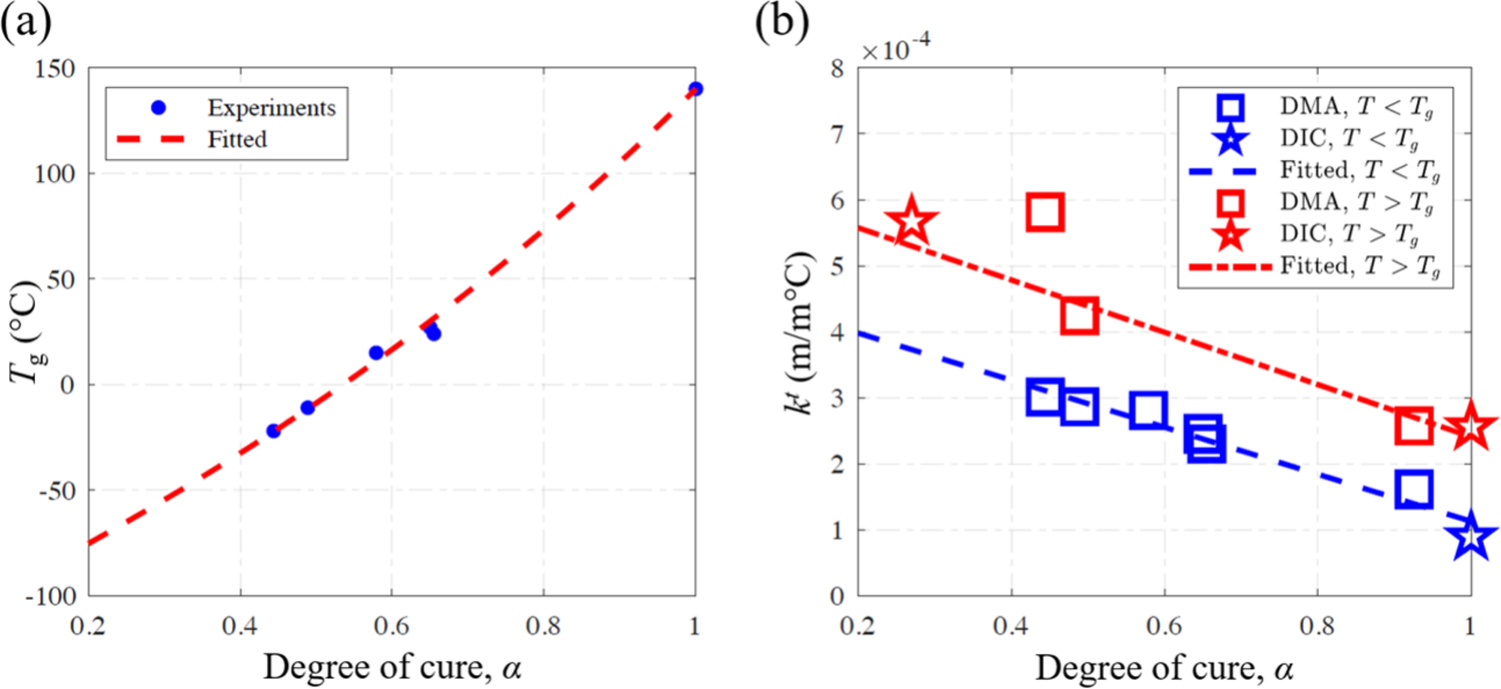
Dependence of (a) glass transition temperature and (b) coefficient of thermal expansion on the degree of cure.

The *k*^t^ of thermosetting polymers is a function of temperature, degree of cure, and the glass transition temperature.^27^ The glass transition temperature, *T*_g_, of DCPD at different degrees of cure was measured by DSC and fitted to DiBenedetto’s equation (Figure 6a)
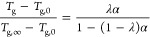
9where *T*_g,0_ and *T*_g,∞_ denote the values of the glass transition temperature of DCPD at α = 0 and 1, respectively, and the fitting parameter λ was determined as 0.7.

*k*^t^ values obtained from both DIC and DMA measurements are presented in Figure 6b, showing consistent trends and values. To incorporate *k*^t^ values into the multiphysics model, the experimental data points were fitted with the following linear functions that describe the variation of the parameter of interest with respect to the degree of cure for temperatures below and above *T*_g_
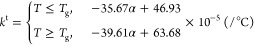
10

**Figure 7:**
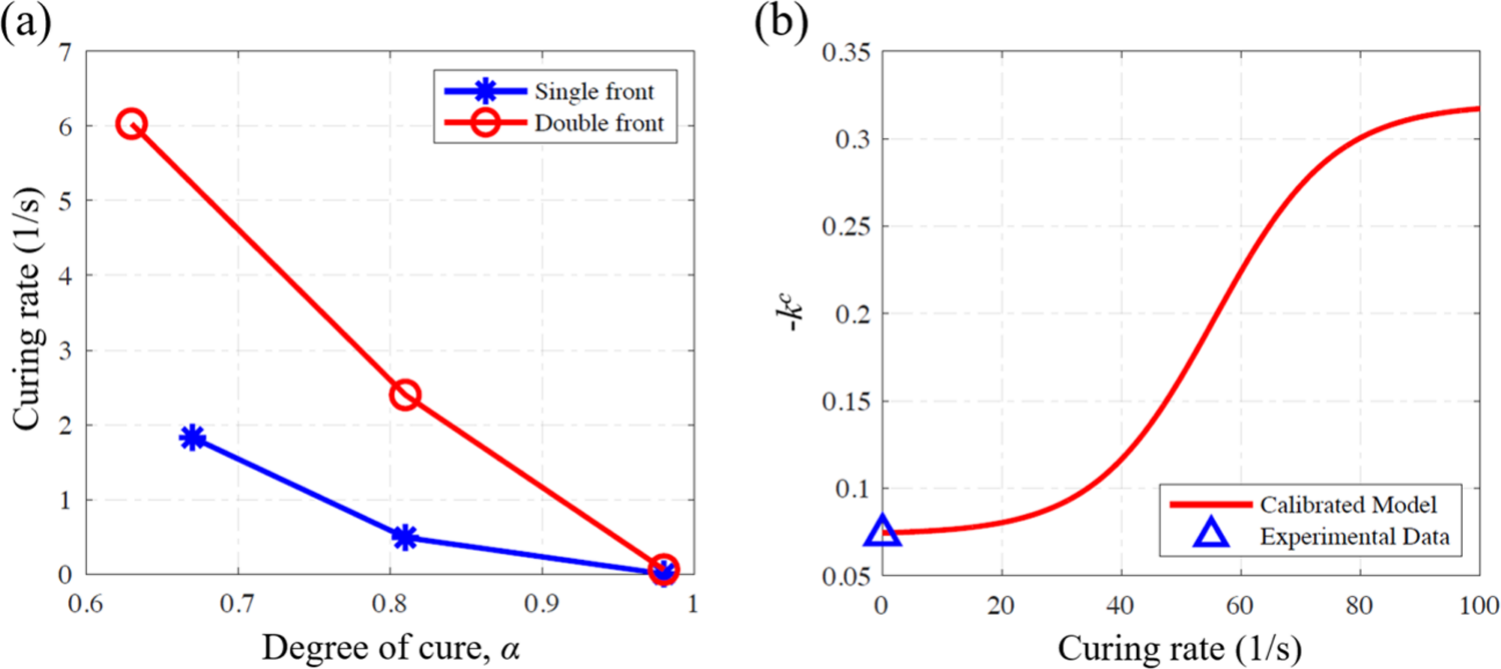
Curing rate-dependent coefficient of chemical shrinkage: (a) different curing rates associated with a single front and converging double fronts; (b) curing rate-dependent *k*^c^, where experimental data was only measured at a low curing rate.

In the present study, the transient chemical reaction and thermal diffusion associated with the moving front impose challenges in isolating the evolution of the chemical shrinkage. We adopted a calibration process to determine the coefficient of chemical shrinkage (*k*^c^), while also aiming to determine the functional form of this parameter. While both constant and cure-dependent *k*^c^ values have been reported in literature,^50,51^ our DIC measurements revealed significant shrinkage in the reacting zone, such as the area exposed to or in the vicinity of the localized heat source (i.e., the tip of the soldering iron). These localized high-temperature regions exhibit accelerated chemical reaction fronts, leading to severe chemical shrinkage. One of the most significant differences between a single front and two merging front cases is the reaction rate, as shown in Figure 7a. Therefore, to capture the excessive shrinkage associated with the higher curing rates at the fronts merging zone, we proposed a curing-rate-dependent *k*^c^, where the *k*^c^ has a higher magnitude (indicating faster and more substantial shrinkage) denoted by *k*_fast_^c^ for higher reaction rates and lower values represented by *k*_slow_^c^ (indicating slower and lesser shrinkage) in slower reactions. We assumed the function form of *k*^c^ to be a sigmoid function shape, expressed as
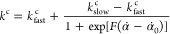
11where *F* and α̇_0_ are nondimensional shape parameters that dictate the shape variation for *k*^c^ over varying (slow to fast) reaction rates. The cure shrinkage was measured by DIC experiments within a small range of slow reaction rates (i.e., temperature does not change hence thermal strain can be excluded, Section 2.3). As such, we obtained the *k*_slow_^c^ = −0.07 by an average value of the experimental data (the calculation process is provided as Supporting Information). The other parameters, including *k*_fast_^c^ and the shape parameters, were obtained by fitting the DIC-measured strains, discussed next. Figure 7b plots the calibrated model in eq 11 with *k*_fast_^c^ equal to −0.32. The parameters *F* and α̇_0_ are taken to be 0.1 and 55.5, respectively. Note that the single experimental data point shown in Figure 7b is obtained by finding the temporal derivative of the experimentally measured degree of cure, presented and discussed in Figure S1 of the Supporting Information document.

### 4.4 Modeling Process-Induced Deformation Fields

**Figure 8:**
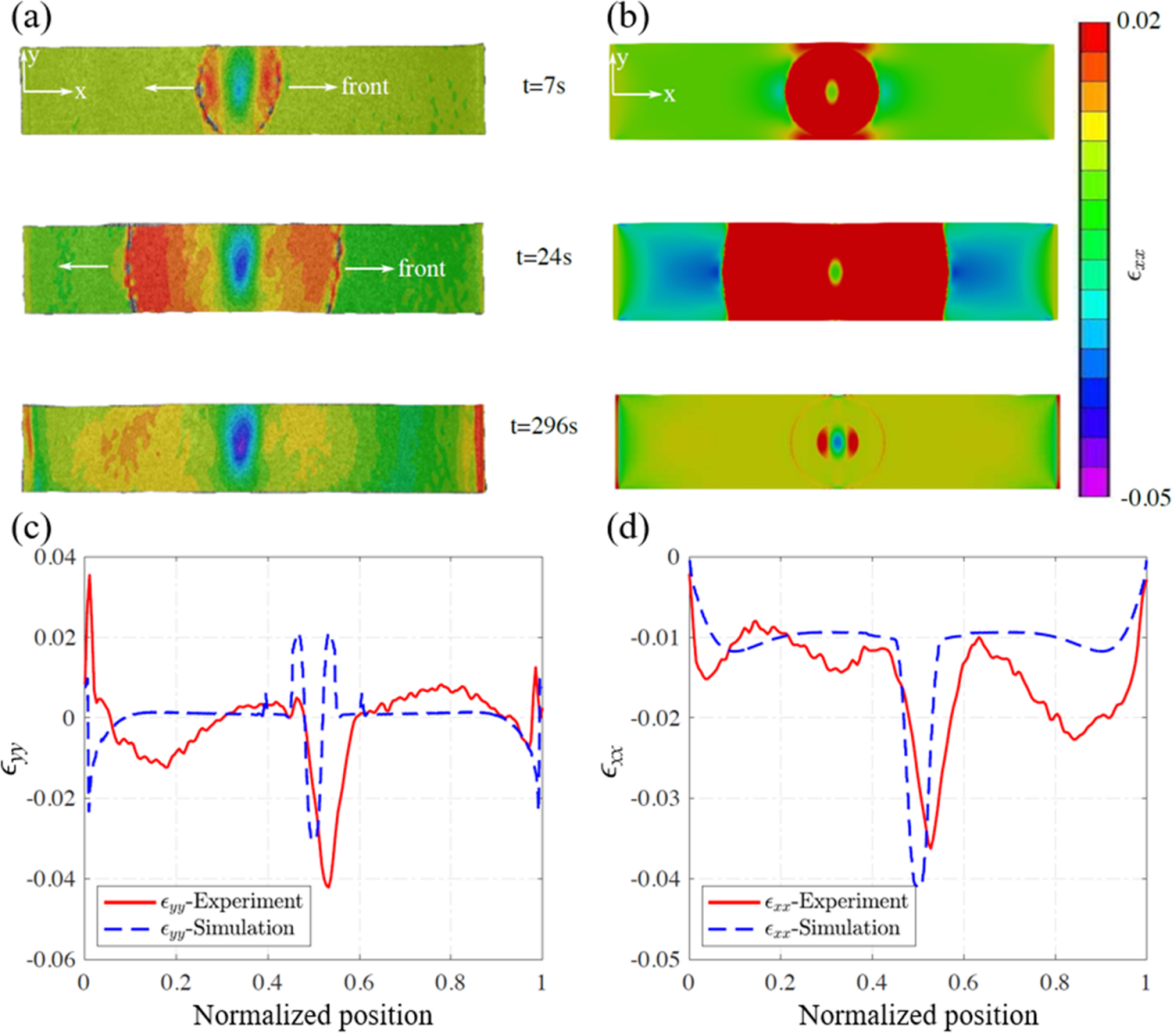
Comparison between measured and simulated strain: (a) measured and (b) simulated strain field ϵ_*xx*_ at different times after the initiation of polymerization. Strain components (c) ϵ_*yy*_ and (d) ϵ_*xx*_ after cool down extracted along the horizontal center line of the specimen.

Figure 8a,b shows a comparison between the DIC-measured and numerically predicted strain fields at different times after the initial thermal triggering of FP, with *t* = 0 marking the onset of polymerization. The positive tensile strain ϵ_*xx*_ obtained in the direction of front propagation follows the front mainly due to the large thermal expansion associated with the hot front. These strain fields are captured by the simulations reasonably well. At the end of the cool-down process, measurements and simulations indicate compressive strains at the center where the front was triggered and tensile strains near the two ends. For a more rigorous comparison, the in-plane strain components ϵ_*xx*_ and ϵ_*yy*_ along the horizontal center line of the specimen are compared in Figure 8c,d. Overall, a reasonably good match is observed, especially with regard to the large compressive strains at the front initiation location.

### 4.5 Mitigating Residual Deformation at Fronts Merging Location by Preheating

**Figure 9:**
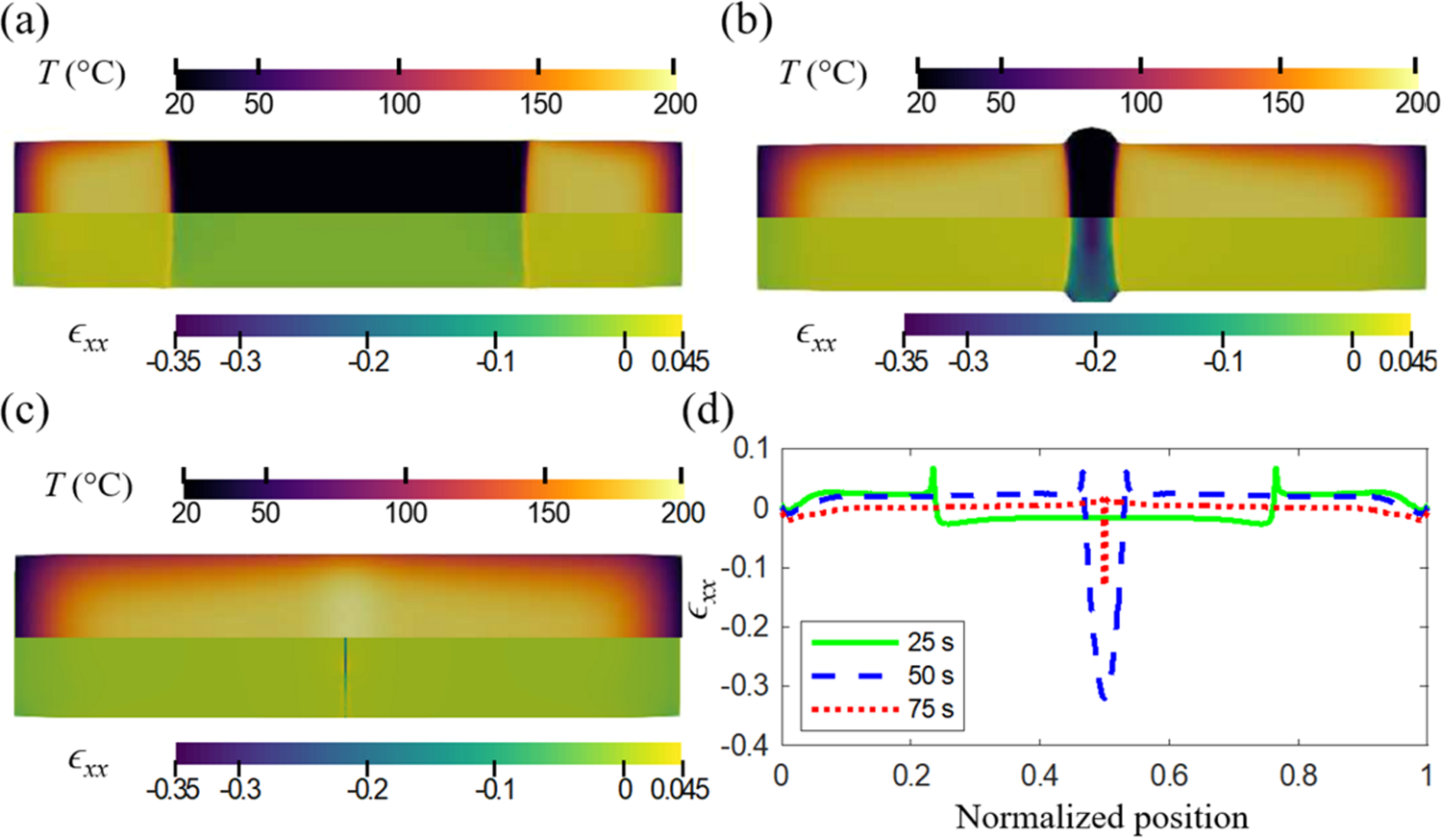
Temperature (shown at the top half of the domain) and strain component ϵ_*xx*_ (shown at the bottom half) distribution at different times after the initiation of the two fronts: (a) *t* = 25 s, (b) *t* = 50 s, (c) *t* = 75 s, (d) comparison of strain ϵ_*xx*_ along the center line at various time instances.

The FP cure time can be reduced by using multiple triggering points.^52^ The merging of multiple fronts leads to temperature spikes (e.g., see Goli et al.^35^ and Centellas et al.^52^), which may introduce a more complex strain field at the front merging area. To study the impact of front merging on residual deformations, we ran simulations with the same domain as in Figure 3 with fronts initiated from the two ends. Figure 9a–c shows the predicted evolution of the temperature and ϵ_*xx*_ strain fields. A significant compressive strain develops in the midsection of the gel piece due to the constraints at two ends and the relatively low stiffness of the uncured gel in the middle, leading to 0.13 residual compressive strains 75 s after initiation.

**Figure 10:**
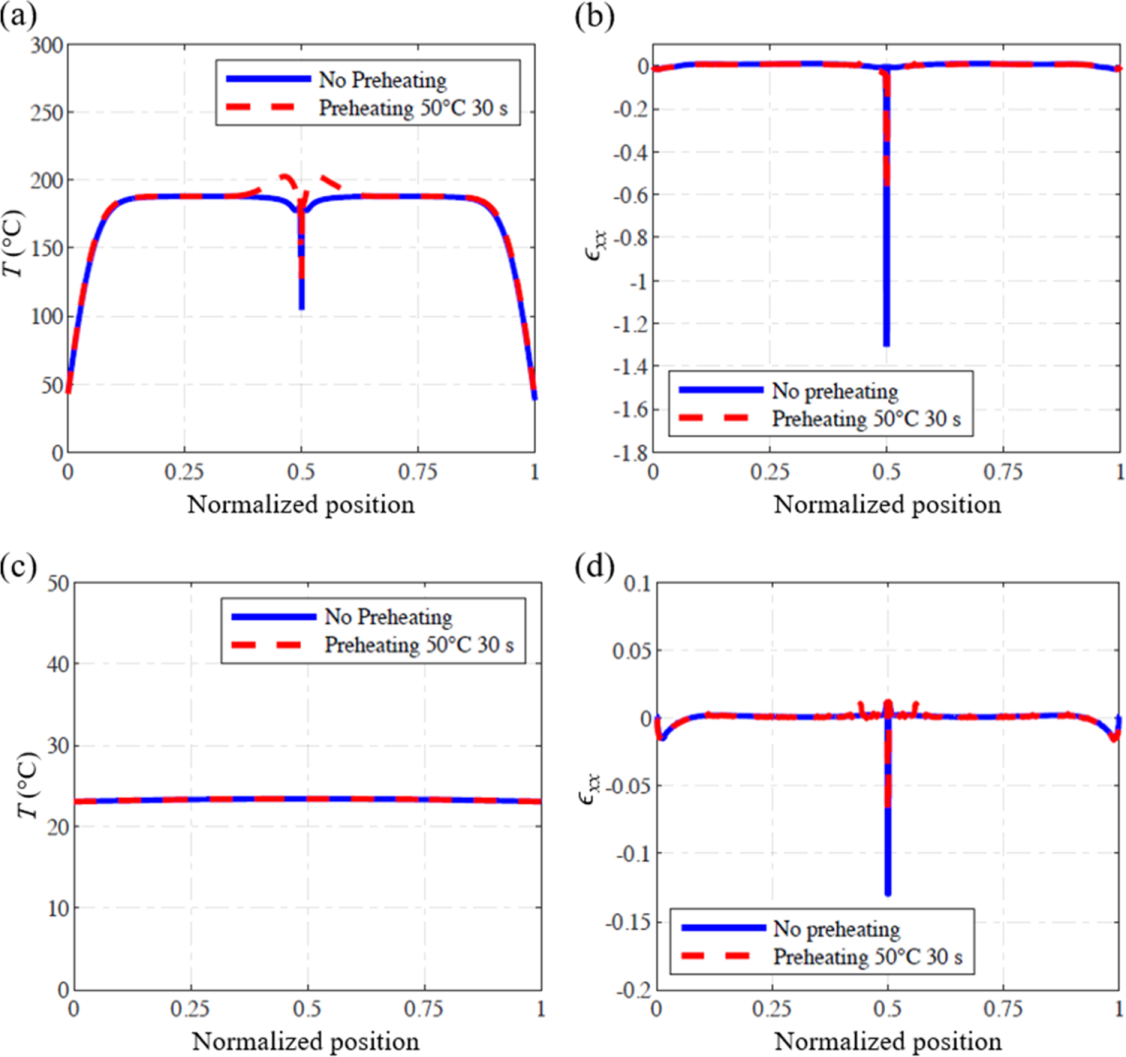
Effect of preheating on local temperature and strains at the fronts merging area: (a) temperature, and (b) residual strain when fronts merge; (c) temperature, and (d) residual strain after the sample cools down, along the axis of FP processed DCPD with and without preheating.

Centellas et al.^52^ reduced the residual strains and decreased the chemical shrinkage in the region where the fronts merged by using a local heat sink (a metallic bar) to eliminate excess heat, thereby substantially reducing the temperature in the front merge zone. Another potential strategy is to concurrently make the material stiffer and slow down the reaction speed in the front merging area. This strategy can be achieved by preheating the region where the reaction fronts are expected to merge. In the specific example discussed here, we choose to preheat a 5 mm wide stripe at the center of the specimen to 50 °C for 30 s. In practice, the preheating can be achieved by both contact and noncontact methods. Figure 10a shows the temperature at the point where the fronts merge with and without preheating. A significantly lower temperature spike is observed when the area local to the merging fronts is preheated. Figure 10b compares the strain components ϵ_*xx*_ along the horizontal center line of the specimen in the two different cases, demonstrating a residual strain reduction of about 50%. This reduction in strain is also observed when the system cools down (Figure 10c,d), further confirming the preheating as an effective approach to reducing the residual deformations in FP processing of DCPD.

## 5 Conclusions

Strain evolution during frontal polymerization in a DCPD system was studied in this work. First, we characterized the cure-dependent thermo-mechanical properties of DCPD via image-based full-field measurements. Elastic moduli and Poisson’s ratios were found to be strongly dependent on the degree of cure, with elastic moduli showing three orders of magnitude difference between partially cured gel and fully cured pDCPD. Poisson’s ratio varied between 0.49 and 0.41 for degrees of cure between 0.28 and 1, respectively. Coefficients of thermal expansion and chemical shrinkage were also measured experimentally as functions of temperature and degree of cure. Chemical shrinkage was found to be highly dependent to the curing rate, causing strong localization of residual strains in accelerated reaction regions, especially where two fronts merge. The multiphysics model created in this work effectively captured the cure-dependent front velocity and the residual strains captured by full-field DIC measurements. Exercising the model revealed that preheating of the monomer (or gel) at the front merging area is an effective method to mitigate residual deformations in these regions.

## Supporting Information Available

Image-based experimental measurements of the coefficient of chemical shrinkage are discussed in the SI document (PDF)
