# Supplementary material for: Residual Strain Development in Rapid Frontally Curing Polymers
Source: ACS Appl Eng Mater. 2024 Oct 31;2(11):2597–607. doi: 10.1021/acsaenm.4c00526 (PMC11590060; doi:10.1021/acsaenm.4c00526)
Supplement: Supplementary file 1 — em4c00526_si_001.pdf [file em4c00526_si_001.pdf]

Supporting Information

Residual Strain Development in Rapid Frontally Curing Polymers

Zhuoting Chen [a,§], Behrad Koohbor [b,§,*], Xiang Zhang [a,*], Leon M. Dean [c,d],

Philippe H. Geubelle [c,e], Nancy R. Sottos [c,d]

a. Mechanical Engineering Department, University of Wyoming, Laramie, WY 82071, USA

b. Department of Mechanical Engineering, Rowan University, Glassboro, NJ 08028, USA

c. Beckman Institute for Advanced Science and Technology, University of Illinois at Urbana-Champaign, Urbana, IL 61801, USA

d. Department of Materials Science and Engineering, University of Illinois at Urbana-Champaign, Urbana, IL 61801, USA

e. Department of Aerospace Engineering, University of Illinois at Urbana-Champaign, Urbana, IL 61801, USA

[§] Authors with equal contribution

* Corresponding Authors:

B. Koohbor (koohbor@rowan.edu), X. Zhang (xiang.zhang@uwyo.edu)

S1. Measurements of the Coefficient of Chemical Shrinkage

The cure dependence of the chemical shrinkage of DCPD was obtained by measuring the strain fields developed in the material over several hours of room temperature curing. In these experiments, a gel sample was allowed to sit freely on a glass plate and cure at a constant temperature of 21±0.5^{o}C while images were acquired over time from its top surface for image correlation purposes. Since the specimen may get some deformations during the handling and since these deformations may not be fully released due to the friction between the specimen and the glass, we only used the data after 2h of measurements to allow the specimen to fully release possible deformations from the handling. The time evolutions of the degree of cure and cure rate

were determined by solving the cure kinetics model (Eq. (1) in the main text) for a constant temperature of 21°C, employing an implicit time integration method (**Figures S1a** and **S1b**).

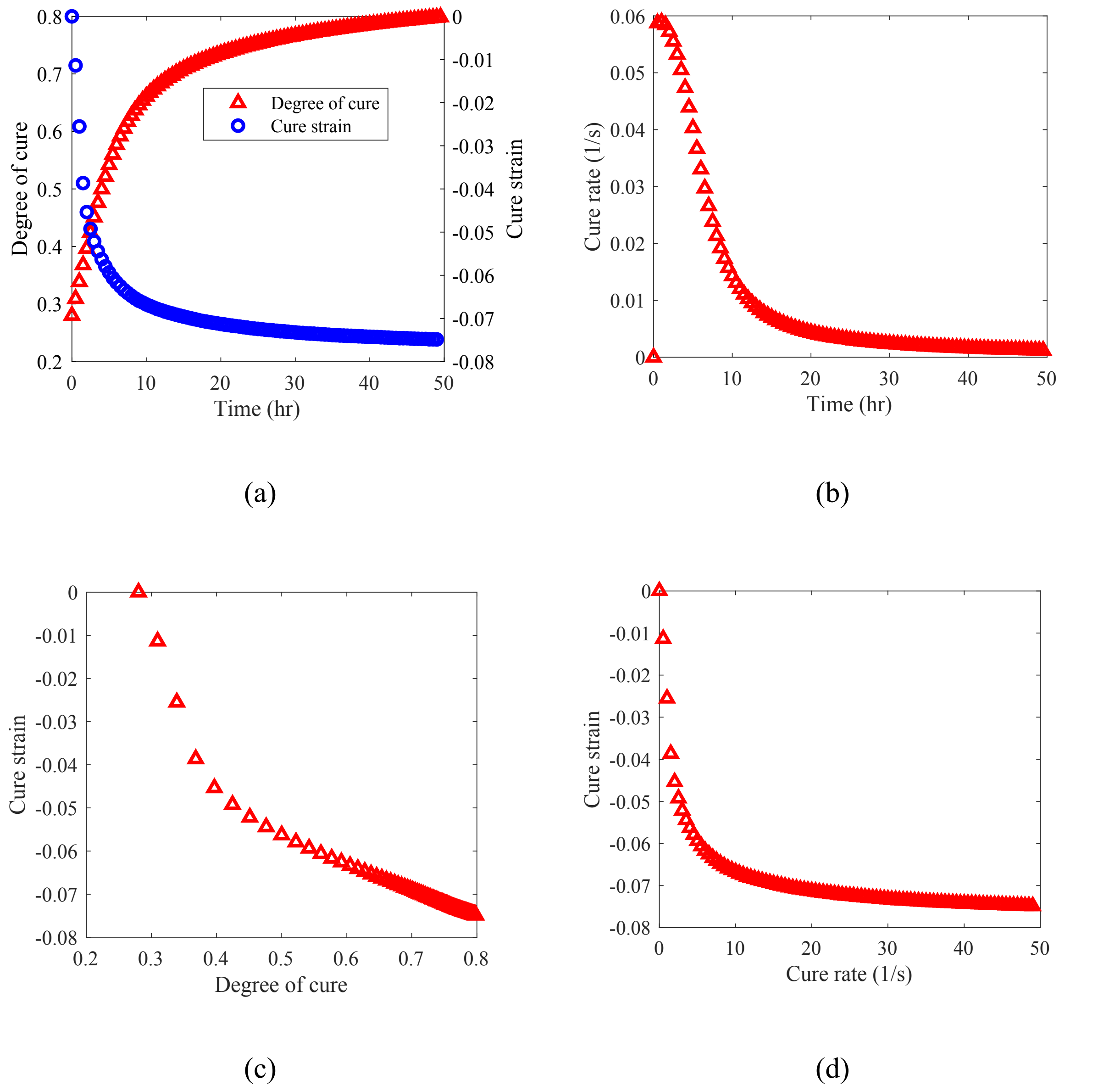

Figure S1. a) Time evolution of the degree of cure and the corresponding in-plane cure shrinkage; b) Time evolution of the cure rate; c) Nonlinear correlation between the cure strain and the degree of cure; d) Nonlinear correlation between the cure strain and cure rate for a DCPD sample cured at room temperature.

The correlation between the shrinkage strain and the degree of cure (**Figure S1c**) and the cure rate (**Figure S1d**) were extracted. Subsequently, the coefficient of chemical shrinkage k^c was determined at varying cure rates (**Figures S2a** and **S2b**) from the evolution of the degree of cure in Eq. (5) of the main text. While this test only covered a relatively low range of curing rate, it pointed to the rate dependence of k^c, which motivated the rate dependent model for k^c used in Section 4.3 of the main text. An averaged k^c was computed from this measurement between t = 2h and t = 49h and used as k_c^{slow} in Eq. (11) since the associated range of curing rate values is small over that period.

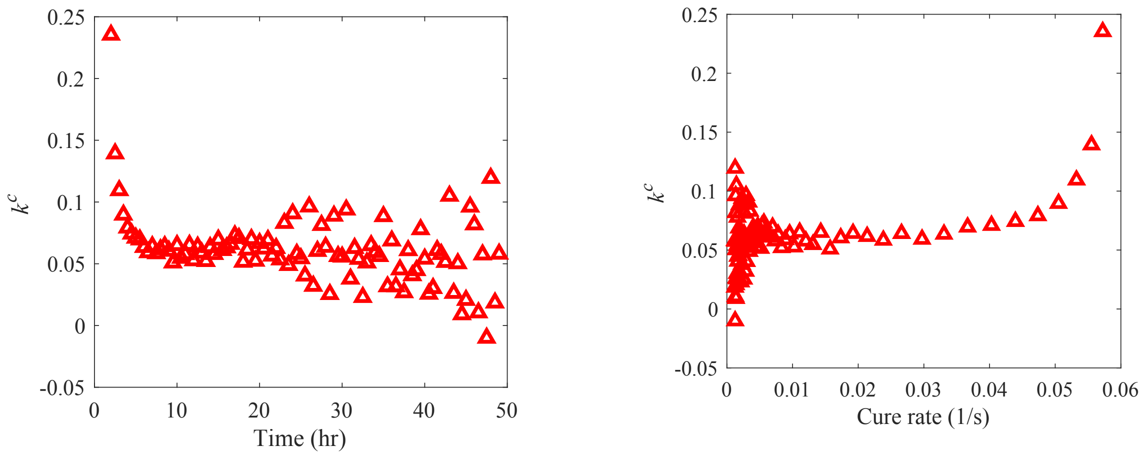

(a) (b)

Figure S2. Coefficient of cure shrinkage k^c *vs.* time (a) and cure rate $d\alpha/dt$ (b).
